# Role of Premycofactocin Synthase in Growth, Microaerophilic Adaptation, and Metabolism of Mycobacterium tuberculosis

**DOI:** 10.1128/mBio.01665-21

**Published:** 2021-07-27

**Authors:** Gopinath Krishnamoorthy, Peggy Kaiser, Patricia Constant, Ulrike Abu Abed, Monika Schmid, Christian K. Frese, Volker Brinkmann, Mamadou Daffé, Stefan H. E. Kaufmann

**Affiliations:** a Department of Immunology, Max Planck Institute for Infection Biology, Berlin, Germany; b Département Tuberculose & Biologie des Infections, Institut de Pharmacologie et de Biologie Structurale, Université de Toulouse, CNRS, UPS, Toulouse, France; c Core Facility Microscopy, Max Planck Institute for Infection Biology, Berlin, Germany; d Core Facility Proteomics, Max Planck Institute for Infection Biology, Berlin, Germany; e Max-Planck-Unit for the Science of Pathogens, Berlin, Germany; f Hagler Institute for Advanced Study at Texas A&M University, College Station, Texas, USA; g Max Planck Institute for Biophysical Chemistry, Göttingen, Germany; Washington University School of Medicine in St. Louis

**Keywords:** mycofactocin, hypoxia, redox cofactor, glucose metabolism, protein aggregation, *Mycobacterium tuberculosis*, lactate metabolism

## Abstract

Mycofactocin is a new class of peptide-derived redox cofactors present in a selected group of bacteria including Mycobacterium tuberculosis. Mycofactocin biosynthesis requires at least six genes, including *mftD*, encoding putative lactate dehydrogenase, which catalyzes the penultimate biosynthetic step. Cellular functions remained unknown until recent reports on the significance of mycofactocin in primary alcohol metabolism. Here, we show that *mftD* transcript levels were increased in hypoxia-adapted M. tuberculosis; however, *mftD* functionality was found likely dispensable for l-lactate metabolism. Targeted deletion of *mftD* reduced the survival of M. tuberculosis in *in vitro* and *in vivo* hypoxia models but increased the bacterial growth in glucose-containing broth as well as in the lungs and spleens, albeit modestly, of aerosol-infected C57BL/6J mice. The cause of this growth advantage remains unestablished; however, the *mftD*-deficient M. tuberculosis strain had reduced NAD(H)/NADP(H) levels and glucose-6-phosphate dehydrogenase activity with no impairment in phthiocerol dimycocerosate lipid synthesis. An ultrastructural examination of parental and mycofactocin biosynthesis gene mutants in M. tuberculosis, M. marinum, and M. smegmatis showed no altered cell morphology and size except the presence of outer membrane-bound fibril-like features only in a mutant subpopulation. A cell surface-protein analysis of M. smegmatis mycofactocin biosynthesis mutants with trypsin revealed differential abundances of a subset of proteins that are known to interact with mycofactocin and their homologs that can enhance protein aggregation or amyloid-like fibrils in riboflavin-starved eukaryotic cells. In sum, phenotypic analyses of the mutant strain implicate the significance of MftD/mycofactocin in M. tuberculosis growth and persistence in its host.

## INTRODUCTION

One-fourth of the human population is infected with Mycobacterium tuberculosis without clinical signs of tuberculosis (TB) disease. Individuals with such latent TB infection have a 10% risk of developing a destructive active disease later during their lifetime (1). Upon inhalation of infectious aerosols, M. tuberculosis reaches human lung alveoli, where it is engulfed by alveolar macrophages. The core of infected macrophages is subsequently surrounded by various immune cells and transforms into a cellular aggregate called granuloma, which acts as a structural barrier restraining dissemination (2). With the clinical progression of TB, a continuum of distinct granuloma types manifests. For example, human latent TB infection is often characterized by solid lung granulomas—containing fewer bacilli—that are surrounded by a fibrotic wall (2–4). These granulomas often include a hypoxic microenvironment wherein both pathogen and host cells respond by undergoing physiological adaptation to sustain their metabolism and survival (2–4).

In M. tuberculosis, the oxidative phosphorylation is the principal component of energy generation and the final electron transfer steps in the respiratory chain require oxygen (5). However, hypoxia, along with reactive nitrogen intermediates, impairs the aerobic respiratory chain of M. tuberculosis and induces a slow- or nonreplicating (dormant) state with characteristic transcriptional induction of DosR-regulated genes and metabolic slowdown (6–9). Under such conditions, M. tuberculosis is, however, believed to use alternate electron acceptors and donors and coordinate several reductases (e.g., nitrate reductase, succinate dehydrogenase/fumarate reductase), associated energy, and redox regulatory pathways. M. tuberculosis has been shown to metabolize glucose through a reverse tricarboxylic acid cycle to generate succinate and maintain a proton gradient across the membrane for hypoxic survival (10, 11). Besides, Watanabe et al. (10) noted that anaerobic-adapted M. tuberculosis secretes lactic acid, although at significantly lower levels than succinic acid; this physiological trait has been speculated to provide another means of membrane energization through reoxidation of reduced nicotinamide cofactors.

MftD (Rv0694, LldD1) is annotated as a putative lactate dehydrogenase sharing 33% sequence homology with Rv1872c (LldD2), but only the latter has been identified as a bona fide enzyme essential for M. tuberculosis growth in lactate (12). Besides, *mftD* is one of the six genes required for the synthesis of mycofactocin, a peptide-derived redox cofactor present in mycobacteria and many other actinobacteria (13). Recently, it has been shown that MftD catalyzes a crucial step forming the penultimate biosynthetic intermediate with an active redox center called “premycofactocin” (14, 15). Cellular functions of mycofactocin, however, remained elusive until recent reports on its significance in primary alcohol metabolism in mycobacteria (16, 17). Protein crystal structure analysis showed that mycofactocin is likely to exchange electrons with NAD^+^ that are bound to alcohol dehydrogenase or other dehydrogenases/oxidoreductases (18). Given that the *mftD* transcript levels were increased in hypoxia-/anoxia-adapted M. tuberculosis (10), either mycofactocin could function as an alternate electron carrier or MftD-mediated lactate dehydrogenase activity could become functionally relevant under oxygen-limited conditions. These possibilities were investigated in the present study. Genetic analysis revealed the dispensable role of *mftD* in lactate metabolism, but it also provided evidence supporting the functionality of mycofactocin in growth, hypoxia adaptation, metabolism, and redox regulation in M. tuberculosis.

## RESULTS

### MftD requirement for M. tuberculosis survival in *in vitro* hypoxia models is glucose dependent.

**FIG 1:**
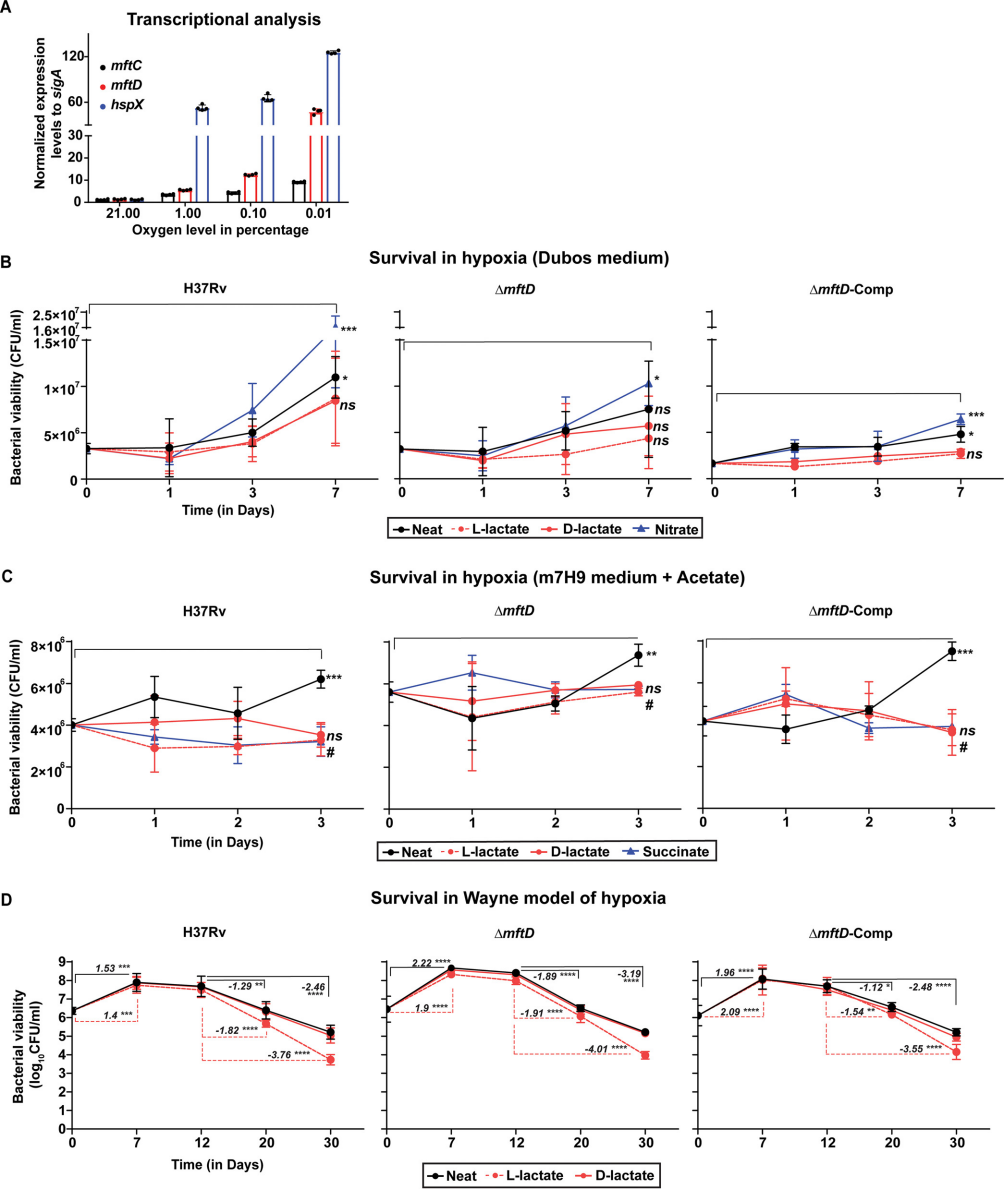
*mftD* is critical for M. tuberculosis adaptation into hypoxia under *in vitro* conditions. (A) M. tuberculosis cells were incubated for 24 h at different oxygen levels. *mftC* and *mftD* transcript levels were increased at lower oxygen levels, indicating their functional relevance. *hspX* expression levels served as control. Data were normalized to *sigA* transcript abundance. (B and C) For hypoxia (1% O_2_) survival experiments, either Dubos medium (B) or modified m7H9 medium containing 0.2% acetate (C) was used. Note Dubos medium is glucose rich (0.75%, wt/vol). Growth medium was supplemented with 10 mM sodium salts of l-lactate (dotted line in red) or d-lactate (solid line in red), or 5 mM nitrate, and 2 mM succinate. Bacterial viability in withdrawn samples was determined by enumerating colonies at a specified time point. Pooled data from three independent experiments were analyzed by two-way analysis of variance (ANOVA)/Šídák’s multiple-comparison test. Statistical significance was determined between initial and final incubation time points. *, *P* < 0.05; **, *P* < 0.01; ***, *P* < 0.001. *ns* denotes nonsignificant. *ns*# denotes nonsignificant in all comparisons. (D) Wayne model of hypoxia. Italicized numerical value represents increase (in positive) or decrease (in negative) in the bacterial growth (in log_10_ CFU), compared at indicated time points. Data from only untreated (neat) and l-lactate (dotted line in red) conditions are highlighted. Data represent the mean ± SD from three independent experiments conducted in replicates, and pooled CFU data were analyzed using two-way ANOVA/Tukey’s multiple-comparison test. Asterisks denote statistically significant differences. ****, *P* < 0.0001; ***, *P* < 0.001; **, *P* < 0.01; *, *P* < 0.05.

Confirming prior observations (10), quantitative real-time PCR (qRT-PCR) analysis revealed that the *mftD* transcript abundance was increased by approximately 55-fold upon exposure of M. tuberculosis to 0.01% oxygen for 24 h (Fig. 1A). Similarly, an 8-fold increase in the transcription of *mftC*, which encodes the enzyme that mediates another important step in mycofactocin biosynthesis, was also noted. The increase in transcription of these two key mycofactocin biosynthesis genes was correlated with decreasing oxygen levels, suggesting their functional relevance, if not that of mycofactocin, in M. tuberculosis survival under hypoxia. To investigate its role in hypoxia adaptation and, in particular, lactate metabolism, an in-frame deletion mutant of *mftD* was constructed and genotyped (see Fig. S1A in the supplemental material). Based on prior experimental evidence (14–17), it is expected that the constructed M. tuberculosis Δ*mftD* is most likely mycofactocin deficient, thereby resembling impaired F_420_ synthesis in a Mycobacterium smegmatis strain lacking either of the F_420_ biosynthesis genes (19). As such, disruption of *mftD* had no impact on M. tuberculosis H37Rv growth on glycerol-supplemented Middlebrook 7H9 (m7H9) medium under well-aerated conditions (Fig. S1B). A genetically complemented strain (herein referred to as Δ*mftD*-Comp) was generated by chromosomally integrating nucleotide sequences comprising *Rv0691c* to *mftD* for comparative assessments.

10.1128/mBio.01665-21.1 FIG S1 Genotypic and phenotypic characterization of M. tuberculosis lacking *mftD.* (A) Confirmation of constructed Δ*mftD* M. tuberculosis mutant by Southern blotting. The target (*mftD*, in red arrow) and flanking genes are shown as solid arrows. Schematic drawn not to scale. For Southern blot analysis, chromosomal DNA from H37Rv wild-type and PCR-confirmed double-crossover mutant was digested with DraIII enzyme overnight. Expected fragment sizes (in base pairs) from H37Rv and Δ*mftD* strains were detected using a PCR-generated probe (solid red line) labeled with digoxigenin (Roche). *In vitro* phenotypes of M. tuberculosis H37Rv and mutant derivatives. (B and G) Growth in liquid medium containing 0.2% glycerol (B) and 10 mM l-lactate (G). Growth curve represents the mean and standard deviation from three independent experiments. (C and D) Bacterial viability under 1% O_2_ was assessed in Dubos medium (C) or m7H9 medium (0.2% acetate) (D). Bacterial viability in withdrawn samples from hypoxia-adapted cultures was determined by enumerating colonies at specified time points. Medium was supplemented with 10 mM sodium salts of l-lactate, d-lactate, nitrate (5 mM), and succinate (2 mM). Pooled data from three independent experiments were analyzed by two-way ANOVA/Šídák'’s multiple-comparison test. Statistical significance was determined between initial and final incubation time points. *, *P* < 0.05; **, *P* < 0.01; ***, *P* < 0.001. *ns* denotes nonsignificant. *ns*^#^ denotes nonsignificant in all comparisons. (E) Wayne model of hypoxia. Italicized numerical value represents increase (in positive) or decrease (in negative) in the Δ*mftD* strain growth (in log_10_ CFU), compared with H37Rv, at indicated time points. Data represent the mean ± SD from three independent experiments conducted in replicates. CFU data were analyzed using two-way ANOVA/Tukey’s multiple-comparison test. Asterisks denote statistically significant differences. *, *P* < 0.05; *ns*, nonsignificant. (F) Bacterial survival in the Wayne culture model using Dubos medium containing 0.1 mM sodium palmitate as a carbon source. Pooled data from two independent experiments performed in duplicate. Download FIG S1, TIF file, 1.9 MB. Copyright © 2021 Krishnamoorthy et al. 2021 Krishnamoorthy et al. https://creativecommons.org/licenses/by/4.0/ This content is distributed under the terms of the Creative Commons Attribution 4.0 International license.

Next, the essentiality of *mftD* for M. tuberculosis *in vitro* hypoxic survival at 1% O_2_ was independently tested in Dubos medium and modified m7H9 medium with acetate (11). While the former is routinely used as a glucose-rich growth base, the latter includes acetate as a sole carbon source representing fatty acids that are believed to be increasingly available for M. tuberculosis during host infection. In both experiments, cultures were incubated only until bacteria reached relatively higher growth (with a statistically significant difference), compared to the initial inoculum. In Dubos medium (Fig. 1B; Fig. S1C), the increment in growth of H37Rv and its mutant derivatives was insignificant until day 3 of incubation at 1% O_2_. However, with the prolonged incubation for 7 days, only the Δ*mftD* strain exhibited delayed growth compared to others. Under the oxygen-limiting condition, nitrate increases the survival of M. tuberculosis replication by acting as an alternate electron acceptor for respiration (20). Consistently, sodium nitrate addition profoundly increased the hypoxic growth of all strains including the Δ*mftD* strain, signifying the specific role of *mftD* in M. tuberculosis adaptation into hypoxia in the absence of nitrate. However, a conflicting outcome was noted in acetate-containing m7H9 medium (Fig. 1C; Fig. S1D), where *mftD* deletion did not affect M. tuberculosis growth. Despite the initial lower inoculum of Δ*mftD*-Comp, the increment in growth of each strain was found significant on day 3 itself, and thus, bacterial incubation was not prolonged further. Nevertheless, the above carbon source (or medium) specific disparity prompted us to assess bacterial survival in closed stirred tubes (or the Wayne model of hypoxia) containing Dubos medium, wherein, the growing M. tuberculosis cells deplete oxygen (inferred through methylene blue decolorization) and sequentially adapt to the onset of hypoxia (nonreplicating persistence [NRP-1] stage) and anoxia (NRP-2 stage) (21, 22). Accordingly, bacterial replication was initially increased, followed by growth stasis and viability loss (Fig. 1D; Fig. S1E). Yet, there were few strain-specific differences. First, methylene blue decolorization (onset of hypoxia) began after 6 days in Δ*mftD* strain-inoculated tubes, whereas it took an additional day in H37Rv and Δ*mftD*-Comp culture tubes. Complete decolorization of methylene blue (anoxia) was evident in all culture tubes between 11 and 12 days of incubation. Thus, the onset of NRP-1 and NRP-2 stages was assumed to be on days 7 and 12 of incubation, respectively. Second, despite use of a comparable inoculum, an 0.8-log_10_ increase in Δ*mftD* strain growth was noted prior to transition into NRP-1, compared with H37Rv. This could be attributed to the early methylene blue decolorization in culture tubes. Finally, during progression through the NRP-2 stage (between days 12 and 30 of incubation), the viability of the Δ*mftD* strain was markedly reduced (−3.19 log_10_) compared with H37Rv (−2.46 log_10_). However, the genetic complementation of the Δ*mftD* strain restored, albeit partially, the phenotype to that of H37Rv level and thereby reaffirmed the likely significance of *mftD* during M. tuberculosis severely hypoxic/anoxic adaptation in glucose-rich Dubos medium. To corroborate whether this Δ*mftD* strain phenotype is glucose dependent, a modified Dubos medium containing 0.1 mM sodium palmitate, instead of 0.75% (wt/vol) glucose, was used for bacterial growth/survival assessment (Fig. S1F). Under such an altered condition, the Δ*mftD* strain had no increased growth fitness or survival deficit during the initial aerobic and later anoxic stages, respectively. Therefore, the critical function of *mftD* in M. tuberculosis survival under a severely limited oxygen condition is most likely glucose specific.

### Extracellular lactate reduces the viability of M. tuberculosis.

*mftD* is annotated as a putative lactate dehydrogenase; however, it was found dispensable for M. tuberculosis to utilize l-lactate (10 mM) as a sole growth substrate under aerobic conditions (Fig. S1G), as shown earlier (12). In contrast, though its dependence on *mftD* is unclear, l-lactate had been found to restrict M. tuberculosis growth under hypoxia (23). Similarly, exogenous succinate had been shown to reduce the viability of M. tuberculosis under hypoxia by impeding the cellular generation and secretion of succinic acid (11). Both succinate and lactate are among the products of fermentation, and thus, it might be that their additive effect on bacterial physiology is similar. To test this notion, the supplementary effect of d- or l-lactate on M. tuberculosis hypoxia survival and associated *mftD* dependency was assessed (Fig. 1B to D; Fig. S1C to E). Compared with the effect of l-lactate, which severely reduced the bacterial growth/viability under oxygen-limited conditions, addition of d-lactate had less or no impact. Importantly, the effects of l-lactate on both H37Rv and Δ*mftD* strains were largely comparable. Moreover, the extracellular addition of succinate resulted in M. tuberculosis growth stasis in acetate medium under 1% oxygen, a phenotype that also served as an experimental control (Fig. 1C; Fig. S1D). In sum, extracellular l-lactate reduces the M. tuberculosis viability with decreasing oxygen levels, underscoring the implication of lactate metabolism for M. tuberculosis microaerophilic survival but confirming that the extracellular lactate effect is nondependent on *mftD* functionality.

### MftD is critical for M. tuberculosis optimal growth in glucose under well-aerated conditions.

**FIG 2:**
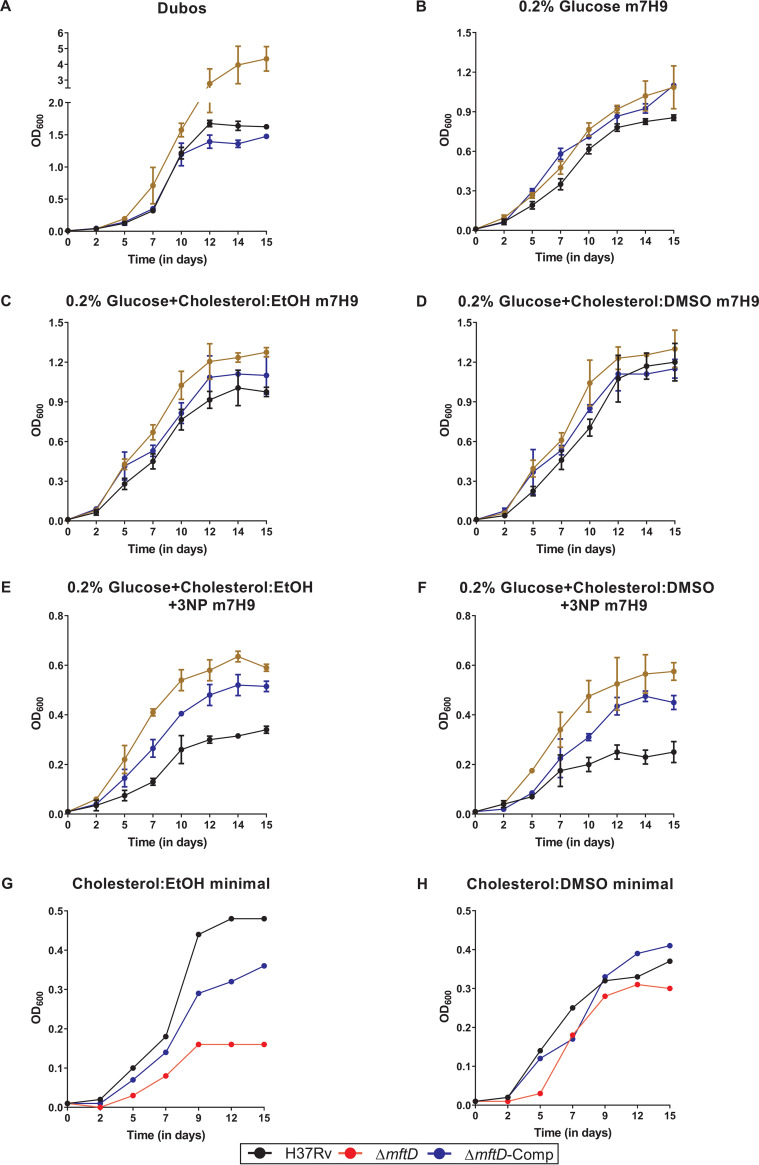
*mftD* disruption increases growth fitness of M. tuberculosis in glucose-containing broth. All growth curve experiments were performed in 490-cm^2^ polystyrene roller bottles with a liquid/air volume ratio of about 1:20 and rotation at 4 rpm. Cell densities (OD) were measured at 600 nm. (A) Growth of Δ*mftD* strain was higher than that of H37Rv and Δ*mftD*-Comp in Dubos medium. According to the Malthusian growth model, which is commonly used to estimate growth rate from during the exponential growth in liquid culture experiments, the estimated doubling time of the Δ*mftD* strain is 3.171 h compared to 4.41 h and 4.644 h for H37Rv and Δ*mftD*-Comp, respectively. (B to D) Growth of bacterial strains in modified m7H9 medium supplemented with 0.2% glucose alone (B), or in combination with cholesterol (0.01%) solubilized either in hot ethanol (cholesterol:EtOH) or hot DMSO (cholesterol:DMSO) (C and D). (E and F) Growth in cholesterol:EtOH (E) or cholesterol:DMSO medium (F) supplemented with 0.2% glucose and 3NP. Data presented in panels A to F are from three independent experiments performed in duplicate. Values shown are means ± standard deviations. (G and H) A representative growth curve of H37Rv and mutant derivatives in minimal medium containing cholesterol:EtOH and cholesterol:DMSO.

As shown above, the initial increase in growth of the Δ*mftD* strain (at the end of the aerobic stage) in the Wayne model is particularly interesting. To validate this, bacterial cells were grown in a roller flask containing Dubos medium (headspace 1:20). Most notably, the Δ*mftD* strain exhibited increased growth fitness, compared with H37Rv and Δ*mftD*-Comp (Fig. 2A). A previous genetic screen had found that mutation in *mftD* (and other mycofactocin biosynthesis genes) restores the growth of the M. tuberculosis isocitrate lyase (ICL) gene mutant—which is otherwise inhibited owing to increased accumulation of toxic metabolic intermediates of methyl citrate and glyoxylate cycles—in solid medium containing cholesterol, fatty acids, and glucose (24). Under such conditions, the restored growth of the ICL mutant in cholesterol could be attributed to the identified glucose-specific increase in fitness of M. tuberculosis upon *mftD* deletion. This reasoning was tested by monitoring bacterial growth in a roller flask with modified m7H9 medium (headspace 1:20) containing 0.2% glucose, 0.01% cholesterol, and/or 0.1 mM 3-nitropropionic acid (3NP), a chemical inhibitor of the ICL enzyme function. Unlike its phenotype in Dubos medium, Δ*mftD* mutant growth kinetics in 0.2% glucose alone was only slightly higher than that of H37Rv (Fig. 2B). However, the increase in growth fitness of the Δ*mftD* strain was more evident in both 3NP-supplemented and -free medium containing cholesterol and glucose as a mixed carbon source (Fig. 2C to F). As noted earlier (16), the presence of ethanol as a solvent restricted the growth of the Δ*mftD* mutant in a medium containing ethanol (EtOH)-solubilized cholesterol (cholesterol:EtOH) (Fig. 2G and H). Therefore, all growth assays were carried out in parallel using dimethyl sulfoxide (DMSO)-solubilized cholesterol (cholesterol:DMSO). Genetic complementation of Δ*mftD*, however, resulted in phenotypes with complete or partial reversion to that of wild-type level. Nevertheless, our results indicate that the growth fitness of the *mftD* mutant is increased in medium containing glucose as a sole or mixed carbon source along with cholesterol under well-aerated conditions, although the underlying mechanism remains unknown.

### MftD function and NADP^+^-glucose-6-phosphate dehydrogenase activity are linked.

**FIG 3:**
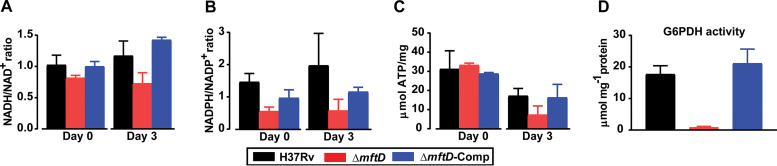
*mftD* is required for redox cofactor recycling and glucose-6-phosphate dehydrogenase activity. Both H37Rv and mutant derivatives were grown to a density of 0.8 to 1.0 (OD_600_). An aliquot of aerobic cell growth (in 0.2% acetate) from each strain was taken (day 0), prior to incubating the culture under hypoxic conditions for 3 days (day 3). (A to C) Samples from aerobic (day 0) and hypoxia-adapted culture in 0.2% acetate containing m7H9 medium were analyzed for the ratio of NADH/NAD^+^ (A) and NADPH/NADP^+^ (B) and the ATP content (C). In parallel, samples of aerobic and hypoxia-adapted culture in Dubos medium were also analyzed, and similar results were obtained. (D) Given the reduced level of NADPH in the Δ*mftD* strain, the activity of G6PDH was measured. Data represent the mean ± SD from at least two independent experiments conducted in replicates. Two-way ANOVA/Tukey’s multiple comparison test on data (A to C) showed no statistically significant differences between the conditions.

Balancing nicotinamide cofactor levels is critical for optimal growth and metabolism. Given the probable redox cofactor functions of mycofactocin (14–17) as well as the increased growth fitness of the Δ*mftD* strain, NAD(H)/NADP(H) and ATP levels in mutant and parental strains were compared using aerobic (day 0, prior to exposure) and hypoxia-adapted (day 3, exposed to 1.0% oxygen for 3 days) culture in Dubos and acetate-containing m7H9 medium. Regardless of the condition, the ratios of reduced and oxidized nicotinamide cofactors were relatively low in the Δ*mftD* strain, compared with those in H37Rv and Δ*mftD*-Comp (Fig. 3A and B). It is known that severely reduced availability of oxygen as a terminal electron acceptor restricts the capacity for NADH reoxidation and thereby increases the NADH/NAD^+^ ratio in M. tuberculosis (10). In agreement, the NADH/NAD^+^ ratio was increased, albeit marginally, in hypoxia-adapted H37Rv and Δ*mftD*-Comp, whereas it remained largely unchanged in the Δ*mftD* strain. These results, however, were not statistically significant. Perhaps, a longer incubation period under 1% hypoxia or other complementary experimental approaches are necessary to confirm the role of *mftD* and/or mycofactocin in redox functions in M. tuberculosis. Additionally, a marginal decline in the intracellular ATP levels was noted particularly in the Δ*mftD* strain, which correlated with a modest decrease in the viability of mutant under oxygen-limiting condition (Fig. 3C).

Cells maintain their NADPH levels by replenishment through metabolic pathways like the pentose phosphate pathway, whose first committed step is mediated by glucose-6-phosphate dehydrogenase (G6PDH) (25). To correlate the slightly reduced NADPH levels noted in the Δ*mftD* strain with diminished activity of NADP^+^-G6PDH, the whole-cell enzyme activity was measured (Fig. 3D). Significantly, the NADP^+^-G6PDH activity was severely reduced in the Δ*mftD* strain compared to other strains, which indicates a possible functional relationship between mycofactocin and NADP^+^-G6PDH in M. tuberculosis.

### The *mftD* mutant is proficient in PDIM/DIM synthesis.

**FIG 4:**
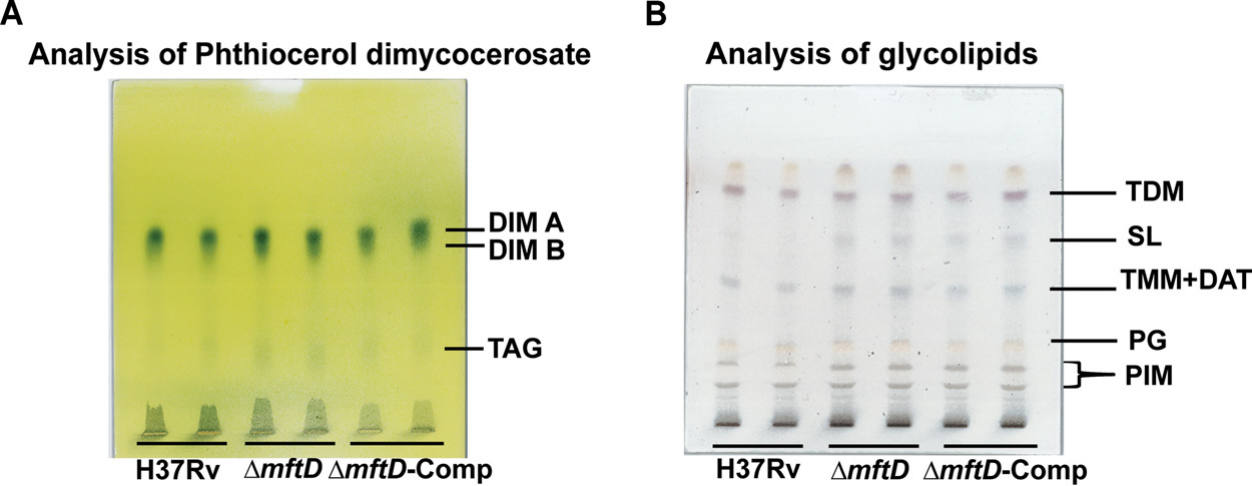
Analysis of phthiocerol dimycocerosate and glycolipids. Total lipid extracts obtained from each strain were analyzed on high-performance thin-layer chromatography (HPTLC) plates run in petroleum ether-diethyl ether (90/10, vol/vol) for phthiocerol dimycocerosate (PDIM/DIM) (A) or in CHCl_3_-CH_3_OH-H_2_O (65/25/4, vol/vol) for glycolipids (B). Abbreviations: TDM, trehalose dimycolate; TMM, trehalose monomycolate; SL, sulfolipid; DAT, diacyl trehalose; PG, phosphatidylglycerol; PIM, phosphatidylinositol mannosides. TLC images are representative of two independent experiments performed in duplicate.

Loss of phthiocerol dimycocerosate (PDIM/DIM) lipid synthesis due to spontaneous mutation in PDIM biosynthesis genes is common during routine *in vitro* propagation, which confers a growth advantage on M. tuberculosis (26, 27). Therefore, we sought to confirm that the increased Δ*mftD* strain growth fitness is not the consequence of permanent loss of PDIM synthesis. To this end, whole-cell lipids from 0.2%-glucose-grown cells were analyzed, and all strains including the Δ*mftD* strain were found to be competent for PDIM production, as observed by DIM-A and -B spots (Fig. 4A). The DIM fractions were enriched through scraping the silica gel (containing these spots) from representative high-performance thin-layer chromatography (HPTLC) plates, and their structural identity was confirmed by nuclear magnetic resonance spectroscopy. In addition, triacylglycerol (TAG) and glycolipid profiles were also similar in both H37Rv and mutant derivatives under the conditions tested (Fig. 4B). Our analysis confirmed, at the least, that there is no permanent loss of PDIM in the Δ*mftD* strains under the conditions tested.

### A subpopulation of *mftD* mutant cells contains extracellular fibril-like structures.

**FIG 5:**
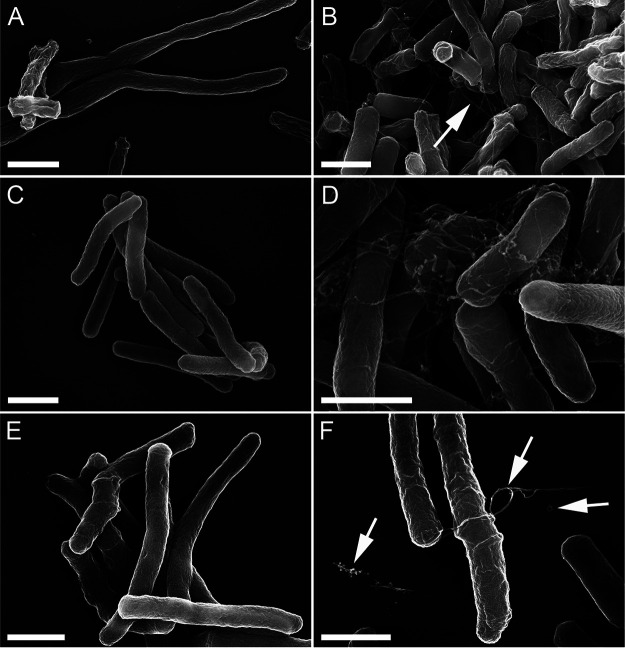
A subpopulation of *mftD* deletion mutant contains extracellular fibril-like structure. (A and B) Scanning electron micrographs of wild-type and Δ*mftD* derivative strains of M. tuberculosis. (C to F) In addition, M. marinum (C and D) and M. smegmatis (E and F) wild-type and Δ*mftD* derivatives were also examined (see also Fig. S2). Indicated arrows highlight the presence of fibril-like structure, a spherical structure, or cell-free aggregates. Size bars represent 1 μm. Images are representative of at least two independent experiments.

Next, we examined the Δ*mftD* cell size and shape using scanning electron microscopy as it exhibited altered growth kinetics under certain conditions. As such, there was no difference between H37Rv and mutant cell size and morphology. Yet, a cell membrane-bound fibril-like structure was detected, albeit not quantitatively, only in a subpopulation of log-phase-growth M. tuberculosis Δ*mftD* (Fig. 5A and B). Detection of such an unusual feature prompted us to additionally examine samples of Δ*mftD* or other mycofactocin biosynthesis gene mutants constructed in Mycobacterium marinum or M. smegmatis mc^2^155. Remarkably, a subpopulation of all mycofactocin biosynthesis mutants had these fibril-like features, which remain undetectable in their respective wild-type samples (Fig. 5C to F; Fig. S2). Besides, some smaller spherical structures were found only in M. smegmatis *mftD* and *mftC* mutants. Of note, the M. smegmatis Δ*mftD* strain did not exhibit any increased growth fitness in Dubos medium or under other conditions tested (data not shown). Thus, fibril-like ultrastructure formation cannot be exclusively linked to increased bacterial growth rate and fitness. Moreover, the genetic complementation did not reverse the phenotype of the M. smegmatis mutant, unlike the M. tuberculosis Δ*mftD*-Comp strain, whose samples were free of such extracellular ultrastructure. An earlier study found a slightly similar extracellular fibril-like structure in aged mycobacterial cultures starved of nutrients (28). However, the occurrence of an ultrastructural feature in mutants here appeared to be distinct and highly specific to loss of function of mycofactocin as the samples of growth-phase-matched wild-type strains were free of any such structure.

10.1128/mBio.01665-21.2 FIG S2 Scanning electron micrographs of mycofactocin biosynthesis mutants showing extracellular fibril-like structures. Scanning electron microscopic (SEM) examination of M. smegmatis derivatives of Δ*mftA* (A), Δ*mftC* (B), Δ*mftE* (C), Δ*mftF* (D), M. tuberculosis Δ*mftD*-Comp (E), and M. smegmatis Δ*mftC*-Comp (F) strains. Indicated arrows highlight the presence of extracellular structure. Size bars represent 1 μm. SEM images are representative of at least two independent experiments. Download FIG S2, TIF file, 1.8 MB. Copyright © 2021 Krishnamoorthy et al. 2021 Krishnamoorthy et al. https://creativecommons.org/licenses/by/4.0/ This content is distributed under the terms of the Creative Commons Attribution 4.0 International license.

Assuming these fibril-like structures are proteinaceous, a “trypsin shaving” technique and liquid chromatography-mass spectrometry (LC-MS) method was sequentially performed to quantitatively analyze the composition of cell surface-associated proteins in nonpathogenic M. smegmatis mc^2^155 and its Δ*mftD* and Δ*mftC* derivatives. Mostly identified were membrane-bound/surface-associated proteins, which ensured the overall experimental goal and data quality. About 313 unique proteins were detected as differentially abundant in either of two mycofactocin biosynthesis mutants compared to the parental strain (Table S2 and Fig. S3). Among the 101 common proteins detected between Δ*mftC* and Δ*mftD* strains, polyphosphate kinase (MSMEG_2391), ATP-dependent CLP protease (ClpP2, MSMEG_4672), and several other hypothetical proteins were found at low abundance. In contrast, NADH dehydrogenase (Ndh) and cytochrome *bc*_1_ oxidase (QcrA, -B, and -C)—one of two terminal oxidases of the electron transport chain—were significantly increased (>1.5-fold) in both mutants. Most notably, the abundance of MSMEG_6242 (and MSMEG_6241) was increased in both mutants similar to its transcriptional induction as reported earlier (16). MSMEG_6242 is an annotated glycerol/alcohol dehydrogenase that interacts with mycofactocin and is implicated in primary alcohol and glycerol metabolism and as a target for pupylation and S-mycothiolation (16, 17, 29–31). Previously, it has been shown that riboflavin cofactor starvation results in the accumulation of flavin-dependent enzymes in structurally and functionally defective forms, which aggregate into amyloid-like fibrils (32, 33). On the same lines, the deficiency of mycofactocin might have resulted in accumulation of MSMEG_6242, in a functional/nonfunctional state, that increased the risk of protein aggregation *in vivo*. Moreover, increased polyphosphate (PolyP) levels (synthesized by polyphosphate kinase) have been found to accelerate protein aggregation (34). The decreased abundance in polyphosphate kinase found in mycofactocin mutants could therefore be perceived as a cellular response to mitigate the increasing proteotoxicity derived from accumulated proteins. Besides, the abundance of MSMEG_0284 (NRH:quinone oxidoreductase [NQO]) was elevated in mycofactocin mutants, which is striking, though its false-discovery rate (FDR) value is higher. MSMEG_0284 was found to share similarities with NQO1 and NQO2 (Fig. S4), which are human riboflavin-dependent enzymes that catalyze reduction of various quinones including ubiquinone and the oxidized form of vitamin K (35, 36). An antagonistic regulation between cofactor-free *apo* NQO1 and reduced 20S proteasome activity had been shown to cause protein aggregation in murine melanoma cells starved of riboflavin (32, 33). Perhaps, increased MSMEG_0284 and decreased ClpP2 abundance in mycofactocin-deficient mutants is possibly involved in a similar mechanism in M. smegmatis. The interpretation is guarded, however, as our evidence is largely indirect and unconfirmed. Nevertheless, the extracellular fibril-like structures in mycofactocin mutant subpopulations might be the consequence of aggregated/misfolded cofactor-free *apo* proteins that are mycofactocin and/or riboflavin dependent.

10.1128/mBio.01665-21.3 FIG S3 Extracellular protein analysis of M. smegmatis and mycofactocin biosynthesis mutants. Trypsin shaving technique and LC-MS-MS method were employed to analyze the outer membrane protein composition of M. smegmatis mc^2^155 and Δ*mftC* and Δ*mftD* mutants. A volcano plot showing the detected proteins with altered abundance. Samples from three independent experiments with two replicates were analyzed. Download FIG S3, TIF file, 0.5 MB. Copyright © 2021 Krishnamoorthy et al. 2021 Krishnamoorthy et al. https://creativecommons.org/licenses/by/4.0/ This content is distributed under the terms of the Creative Commons Attribution 4.0 International license.

10.1128/mBio.01665-21.4 FIG S4 Sequence alignment analysis of MSMEG_0284, human NQO1 and NQO2. On performing protein sequence homology using Clustal Omega, MSMEG_0284 (GenBank accession no. ABK70726.1) was found to share 31% and 34% identities with human NQO1 (NCBI reference sequence no. NP_000894.1) and NQO2 (NCBI reference sequence no. NP_000895.2), respectively. Amino acid residues with sequence similarities and secondary structure elements of quinone reductase (based on the work of Deller et al. [36]) are indicated. α helices, black on gray background; β sheets, white on black background; loops, black on white background. Download FIG S4, TIF file, 1.3 MB. Copyright © 2021 Krishnamoorthy et al. 2021 Krishnamoorthy et al. https://creativecommons.org/licenses/by/4.0/ This content is distributed under the terms of the Creative Commons Attribution 4.0 International license.

10.1128/mBio.01665-21.7 TABLE S2 Cell surface protein analysis of M. smegmatis mc^2^155 and Δ*mftC* and Δ*mftD* mutant derivatives. Download Table S2, XLSX file, 0.2 MB. Copyright © 2021 Krishnamoorthy et al. 2021 Krishnamoorthy et al. https://creativecommons.org/licenses/by/4.0/ This content is distributed under the terms of the Creative Commons Attribution 4.0 International license.

### MftD function impacts the growth of M. tuberculosis in murine models.

**FIG 6:**
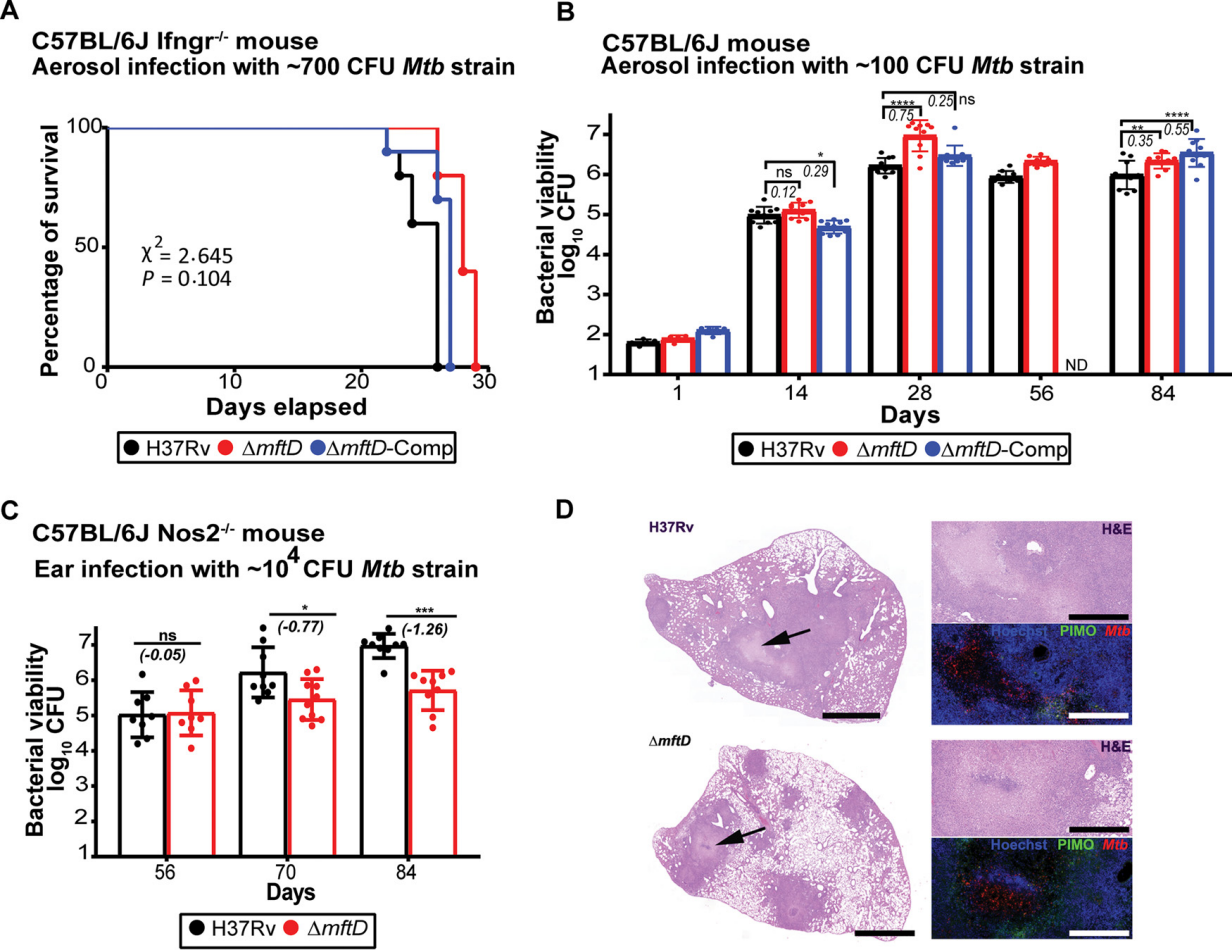
*mftD* function is required for M. tuberculosis normal growth and survival in murine TB models. (A) C57BL/6J Ifngr1^−/−^ mice were aerosol infected with ≈700 CFU H37Rv and mutant derivatives; survival of infected mice was recorded. Data were analyzed by log rank test, and there was no statistically significant difference between groups (*P* = 0.1038). (B) C57BL/6J mice were aerosol infected with ≈100 CFU of M. tuberculosis strains. Pulmonary bacterial burden was monitored at indicated time points. Data sets presented are from 2 independent experiments (total *n* = 9 to 10). Values shown are means ± standard deviation (SD). Italicized numerical values represent a growth increment of mutant (in log_10_ CFU), compared to H37Rv. ND denotes not done. Statistical significance was determined using two-way ANOVA with multicomparison and Tukey’s posttest. Asterisks denote statistically significant differences; ns denotes nonsignificant differences. ****, *P* < 0.0001; **, *P* < 0.01; *, *P* < 0.05. (C) Δ*mftD* strain growth and survival in C57BL/6J Nos2^−/−^ mice with hypoxic necrotizing lung lesions were assessed. Onset of central necrosis and hypoxia in lung lesions on day 56 was evident. Lung CFU data (means ± SD) from two independent experiments (total *n* = 9 to 10) are shown. Italicized numerical value (negative) represents reduction in the mutant growth (in log_10_ CFU), compared with wild-type H37Rv. Pooled data from two independent experiments were analyzed using two-way ANOVA/Šídák’s multiple-comparison test. *, *P* < 0.05; ***, *P* < 0.001. (D) Hematoxylin and eosin (H&E) staining and immunofluorescence detection of cell viability (Hoechst 33342), M. tuberculosis (Mtb), or hypoxia marker PIMO. Micrographs of an H&E-stained section of whole left lung lobe are presented in the left panel. Scale bar represents 2.5 mm. Magnified images show staining of Hoechst (blue), M. tuberculosis (red), and PIMO (green) in lung lesion. Scale bar represents 1 mm.

To determine whether *mftD* deletion alters M. tuberculosis virulence, highly susceptible interferon gamma (IFN-γ) receptor knockout mice (C57BL/6J Ifngr1^−/−^) were aerosol infected with either H37Rv or mutant derivatives, and survival was recorded (Fig. 6A). Mice infected with the Δ*mftD* strain survived for a slightly higher median time of 28 days, compared with H37Rv-infected mice with 26 days of survival time, indicating the dispensability of *mftD* in M. tuberculosis virulence in the absence of IFN-γ signaling.

Subsequently, the significance of *mftD* in M. tuberculosis growth and survival in immunocompetent C57BL/6J mice was assessed. To this end, mice were aerosol infected, and sacrificed at different time points following infection, and the bacterial burdens in their lungs and spleens were enumerated. Initially, there were no notable strain-specific differences in the bacterial loads (Fig. 6B; Fig. S5A). However, the Δ*mftD* strain pulmonary growth fitness was increased between days 14 and 28 postinfection, compared to the H37Rv kinetics. Such an increase in Δ*mftD* strain growth is reminiscent of its *in vitro* phenotype in glucose-containing medium. Thereafter, both H37Rv and Δ*mftD* strain viable counts reached a plateau in lungs of infected mice during the late stage of infection. Besides, there were no differences between H37Rv and Δ*mftD* strains in their ability to persist in lungs, i.e., there was no bacterial survival deficit until the end of the experiment. However, unlike the other two strains tested, Δ*mftD*-Comp growth fitness was higher between days 28 and 84, indicating only partial functional restoration of the mutant phenotype, and thus, this strain was excluded in the subsequent experiment. Failure to achieve complete complementation is a concern. It could be due to improper expression of *mftD* or due to its impact on the functional coordination of other mycofactocin biosynthesis genes depending on experimental conditions.

10.1128/mBio.01665-21.5 FIG S5 Impact of *mftD* disruption on M. tuberculosis growth in murine TB models. (A and B) Splenic counts (in log_10_ CFU) of H37Rv, Δ*mftD*, or Δ*mftD*-Comp strains in aerosol-infected C57BL/6J mice (A) and intradermally infected C57BL/6J Nos2^−/−^ hypoxia mouse model (B). Panel A is denoted only with values that are statistically significant. Pooled data from two independent experiments were analyzed using two-way ANOVA/Šídák'’s multiple-comparison test. *, *P* < 0.05; **, *P* < 0.01. (C and D) Total number of lesions (C) and number of necrotic lesions (D) among the C57BL/6J Nos2^−/−^ mouse groups infected with M. tuberculosis strains. Data from a representative experiment (total *n* = 5) were analyzed using two-way ANOVA with multiple comparison and Tukey’s posttest. Statistical significance was determined between H37Rv and mutant derivatives at indicated time points. Download FIG S5, TIF file, 1.8 MB. Copyright © 2021 Krishnamoorthy et al. 2021 Krishnamoorthy et al. https://creativecommons.org/licenses/by/4.0/ This content is distributed under the terms of the Creative Commons Attribution 4.0 International license.

Widely used murine models (e.g., C57BL/6 or BALB/c) do not recapitulate the human TB pathology. Previously, in our group, a C57BL/6J Nos2^–/–^ mouse model presenting granuloma that includes central necrosis, hypoxia, and caseation has been established with an intradermal M. tuberculosis infection and transitory blocking of tumor necrosis factor alpha (TNF-α) function (37–39). This mouse model was used to assess survival of Δ*mftD* and H37Rv strains in hypoxic lesions (Fig. 6C). On day 56 postinfection, both bacterial burdens and lung pathologies of Δ*mftD* strain- and H37Rv-infected groups remained almost identical. With the onset of hypoxia and progressing infection, however, the Δ*mftD* strain grew more slowly and its viable counts were 0.77- and 1.26-log_10_-CFU lower than those of H37Rv at days 70 and 84 postinfection, respectively. A corresponding reduction in the splenic bacterial count was also noted (Fig. S5B). Nevertheless, numbers of total or hypoxic pimonidazole hydrochloride (PIMO)-positive and necrotic granulomas between the mutant- and H37Rv-infected groups were comparable at days 56 and 84 postinfection (Fig. 6D; Fig. S5C and D). M. tuberculosis possesses various pathogenic strategies that control its ability to actively replicate or maintain quiescence in host lungs for an extended period. Our *in vivo* data therefore implicate a critical role for MftD in M. tuberculosis optimal growth and survival in murine lung tissues.

## DISCUSSION

Although the MftD-mediated step in synthesis of mycofactocin is aerobic (14, 15), our results revealed a role for *mftD* in M. tuberculosis persistence under hypoxic/anoxic conditions. This contrasting oxygen-specific scenario corresponds to the intracellular lifestyle of M. tuberculosis transiting between aerobic growth and microaerophilic/anaerobic persistence within the tuberculous granuloma. The *mftD*-encoded product catalyzes the crucial step in formation of premycofactocin—a penultimate biosynthetic intermediate, which could oxidize NADH, and its calculated midpoint potential is ≈−255 mV (14). Another noncanonical cofactor is F_420_, which is generated through the aerobic route with relatively lower redox potential in mycobacterial species but facilitates M. tuberculosis microaerophilic adaptation (19, 40–42). F_420_ cofactor recycling in methanogenic (anaerobic) archaea is achieved by F_420_ reductase (Fno), which transfers electrons between F_420_ and NADPH pools. As such, mycobacterial genomes lack an *Fno* ortholog. Instead, the F_420_ regeneration in mycobacterial species is mediated by F_420_-dependent G6PDH (Fgd1 and Fgd2), which oxidizes glucose-6-phosphate with coupled reduction of F_420_ into F_420_H_2_ (43, 44). Unlike F_420_, the electrochemical properties of mature mycofactocin and the biochemistry of redox interactions between mycofactocin and dependent enzymes remain unknown. However, there are a few hints on mycofactocin cofactor regeneration. First, the diminished NADP^+^-G6PDH activity in the Δ*mftD* strain might indicate that M. tuberculosis NADP^+^-G6PDH (Rv1121 or Rv1447c), similar to other identified dehydrogenases/oxidoreductases (18), involves mycofactocin as an external redox exchange system to mediate recycling of NADP^+^ to NADPH for its turnover and concomitant mycofactocin regeneration. Second, the functions of MftD and Fno are predicted to be analogous (13), implying possible similarity between F_420_ and mycofactocin recycling under oxygen limitation. Reinforcing this view, *mftD* (and *mftC*) is among the genes induced upon the activation of pyruvate metabolism, which is critical for the regeneration of reduced cofactors by fermentation (9, 23). As the role of *mftD* in M. tuberculosis hypoxic/anoxic survival is recognized in this study, it is tempting, therefore, to speculate that mycofactocin might act as an alternate electron carrier, similar to F_420_, during microaerophilic respiration or fermentation. While these possibilities require further confirmation, it is also important to ascertain the functional relevance of *mftD* or other mycofactocin biosynthesis genes in metabolic adaptation during M. tuberculosis subsistence on alternative carbon sources or lactate metabolism under hypoxia. Resulting insights will allow comprehensive understanding of mycofactocin-associated redox regulation/pathways as well as their significance in M. tuberculosis hypoxia adaptation, a fundamental feature associated with latent TB infection that still remains unclear.

Glucose-specific increase in growth fitness of the Δ*mftD* strain is remarkable though it occurs only at a high concentration, which is unlikely to be pathophysiologically relevant. As such, there is no established association between *mftD* function and glucose metabolism except the diminished whole-cell G6PDH activity noted in the mutant and its implicated role in pyruvate metabolism (23). It is equally uncertain whether *mftD* disruption altered the respiration state even though *mftC*- and *mftD*-encoded products have been predicted as a component of the mycobacterial respiratory system (5). Our previous investigation showed that deletion of *mftC* is unlikely to alter the respiration (inferred through measuring oxygen consumption rate [OCR]) in adherent M. tuberculosis/M. smegmatis cells measured using a real-time extracellular flux analyzer (16). In contrast, disruption of succinate dehydrogenase, a proposed regulator of respiration, increased the OCR (assessed in a batch culture bioreactor) without affecting the rate of M. tuberculosis exponential growth owing to an imbalance in the redox state of the menaquinone pool (45). Other studies have demonstrated that the deactivation of *whiB3*, a redox sensor protein-encoding gene, reduced OCR in M. tuberculosis when pyruvate served as the substrate (assessed in adherent cells using a real-time extracellular flux analyzer) but increased the bacterial growth fitness in acetate-containing medium (46, 47). Thus, knockout mutants of genes even with a recognized role in energy and/or redox-associated metabolism often result in variable phenotypes owing to functional redundancies in the mycobacterial multibranched respiratory chain or differences in experimental design and readouts (5, 9–11, 45–47). Hence, genetic and growth phenotypic analysis alone is inadequate to establish *mftD* function in M. tuberculosis respiration. It is possible that disruption of *mftD* could affect multiple functions, which could be identified by multi-omics approaches including nontargeted and targeted metabolomics analyses. Yet, the altered NADH/NAD^+^ ratio and increased Ndh/ubiquinol-cytochrome *c* reductase subunit abundance in Δ*mftD* derivatives of M. tuberculosis and M. smegmatis, respectively, perhaps imply a perturbed state of respiratory chain function upon the loss of *mftD* or mycofactocin-associated function. On the other hand, pyrroloquinoline quinone (PQQ), also a peptide-derived cofactor, enables the function of quinoprotein glucose dehydrogenases, which oxidize d-glucose to d-gluconate and feed electrons to ubiquinol oxidase via ubiquinone or menaquinone in the respiratory chain (48). PQQ and mycofactocin share many similarities (13); however, whether mycofactocin could mediate an identical role in glucose oxidation and coupled respiration or redox regulation in mycobacteria is the subject of future investigation.

Riboflavin is the precursor of flavin mononucleotide (FMN) and flavin adenine dinucleotide (FAD). Intriguingly, MftD is a flavoprotein that has been shown to reduce FMN during mycofactocin synthesis (14). Cooccurrence of extracellular fibril-like structures and altered abundance of certain proteins in mycofactocin-deficient mycobacteria indicated the possibility of aggravated protein aggregation, thereby resembling riboflavin-starved eukaryotic cells (32, 33). Such reasoning is further strengthened by the detection of a fibril-like structure typically at a cell pole wherein protein aggregates tend to accumulate in M. tuberculosis (49–51). However, the underlying reasons for the presence of fibril-like structures only in a subpopulation remain unclear, and the following could account for this observation. During cell division, protein aggregates pass on to progeny through asymmetrical partitioning and usually result in a phenotypically heterogeneous population (49–51). Alternatively, these structures are destined to be secreted and might not be cell bound throughout the cell cycle. In addition, the detection of cell-free aggregates suggests that a proportion of cells is lysed owing to increased proteotoxicity.

Although succinate and lactate efflux mechanisms in some bacteria have been found to be similar (52, 53), we could not establish whether exogenous l-lactate addition impairs hypoxia-induced cellular generation and efflux of lactic acid and consequently affects the M. tuberculosis membrane potential and viability as in the case of succinate (11). Extracellular lactic acid could diffuse across the membrane, dissociate, and release protons to increase intracellular acidification and compromise bacterial viability under oxygen-limited conditions (54, 55); this possibility could not be eliminated in our experiments. Nonetheless, it is clear that l-lactate supports aerobic growth of M. tuberculosis (12, 23), but it exhibits bacteriostatic or bactericidal effects with the depletion of oxygen as shown here. TB granuloma microenvironments present abundant lactate with fluctuating oxygen levels (56, 57), and in such a milieu, l-lactate could directly modulate the replication dynamics of M. tuberculosis ranging from growth inhibition to resumption. With the increasingly known effects on host metabolism and anti-TB immune responses (58–60), l-lactate therefore likely influences the physiology of host-M. tuberculosis cross talk and disease progression.

While our study offers several strong clues, the multiple and apparently unrelated phenotypes of the *mftD* mutant cannot yet provide a comprehensive insight into the mechanism of mycofactocin-associated processes in mycobacteria. Regardless of this limitation, identifying the functional relevance of the mycofactocin cofactor under diverse conditions is substantial as cellular biochemical processes can principally be perceived to be mediated only by a few known cofactors and their dependent enzymes. Our study outcome therefore constitutes a basis for future research investigating questions related to mycofactocin in mycobacteria and other mycofactocin-producing bacteria, such as the following: what are the implications of mycofactocin in bacterial metabolism and physiology? Which enzymes are mycofactocin dependent? How does mycofactocin interact with its dependent enzymes?

## MATERIALS AND METHODS

### Bacterial strains and growth assays.

M. tuberculosis H37Rv (American Type Culture Collection, 27294^TM^) and mutant derivatives were grown in complete Middlebrook 7H9 (m7H9) broth (Becton, Dickinson [BD]) supplemented with albumin-dextrose-catalase enrichment (BD), 0.2% glycerol, and 0.05% tyloxapol or on m7H11 agar (BD) containing 10% (vol/vol) oleic acid-albumin-dextrose-catalase enrichment (BD) and 0.2% glycerol. Bacterial cultures of 10-ml volume were routinely grown in Nalgene square polyethylene terephthalate glycol (PETG) bottles (30 ml; Thermo Fisher Scientific) with orbital shaking (100 rpm) at 37°C. All other chemicals were from Sigma-Aldrich/Fisher Scientific unless otherwise specified.

For the *in vitro* hypoxia model, either Dubos medium (BD) or modified m7H9 medium containing 0.2% acetate, 0.085% sodium chloride, 0.05% tyloxapol, and 0.5% bovine serum albumin (BSA) (11) was used. Bacterial inocula were grown into mid-log phase in complete m7H9 medium. Bacterial strains were further adjusted to the optical density at 600 nm (OD_600_) of 0.5 in a fresh appropriate growth medium prior to incubation at 37°C, for a specified time, in a hypoxia chamber (Coy Laboratory) where oxygen and carbon dioxide levels were maintained at 1% ± 0.2% and 5% ± 0.2%, respectively. The hypoxic state was ensured using an inbuilt system and resazurin indicator strips. At specified time points, viable counts were enumerated from collected samples. For the Wayne model, bacterial strains were grown in Dubos medium supplemented with 0.5% BSA, 0.085% sodium chloride, and 0.75% dextrose or 0.1 mM sodium palmitate. Bacterial culture was grown in sealed tubes with slow magnetic stirring (120 rpm) with a liquid/air volume ratio of 0.75:1 as previously described (21, 22). The supplementary effect of 10 mM sodium l- and d-lactate, 5 mM sodium nitrate, and 2 mM sodium succinate was tested.

For growth curves presented in Fig. 2A to F, Dubos or modified m7H9 medium was used containing 0.5% BSA, 0.085% sodium chloride, 0.2% glucose, and/or 0.01% cholesterol. Where indicated, 0.1 mM 3-nitropropionate (3NP) was used. Carbon utilization assays in Fig. 2G and H were performed using minimal medium as described earlier (16). Cholesterol (0.01%) was solubilized in 0.5% hot ethanol or 1% hot DMSO. Other carbon sources tested were 10 mM sodium l-lactate and 0.2% glycerol. Under each condition, bacterial cultures were grown in 490-cm^2^ polystyrene roller bottles (Corning) with a liquid/air volume ratio of about 1:20 and rotation at 4 rpm at 37°C.

Infection stocks were prepared from mid -log-phase M. tuberculosis cultures. For CFU determinations, serial dilutions were performed in phosphate-buffered saline (PBS)–0.05% Tween 80 and plated onto m7H11 agar. Plates were incubated at 37°C for 4 to 5 weeks prior to CFU counting. All experiments involving M. tuberculosis were carried out in biosafety level 3 laboratories.

### Genetic manipulation.

Unmarked deletion of the *mftD* gene was made by a two-step allelic-exchange method reported earlier (61). A knockout plasmid was constructed with a homologous genomic region flanking an in-frame fusion of the first 2 codons to the last 6 codons of *mftD*, to replace the native copy of the gene. The genomic deletion was confirmed by PCR and Southern blotting. The genomic region comprising *Rv0691c* to *mftD* genes was PCR amplified and cloned into the HindIII site of pMCpAINT (62) carrying a kanamycin resistance marker for genetic complementation.

### Transcriptional analysis.

Twenty-milliliter Dubos medium-grown log-phase M. tuberculosis H37Rv culture samples were aliquoted and incubated at 37°C for 24 h in a hypoxia chamber where oxygen and carbon dioxide levels were maintained at indicated values and 5% ± 0.2%, respectively. RNA was prepared as described previously (16). First-strand cDNA synthesis was performed with the iScript cDNA synthesis kit for qRT-PCR (Bio-Rad). Real-time quantitative PCR was carried out on the Roche LightCycler 480 system with iTaq Universal Probes Supermix (Bio-Rad) using the Molecular Beacons system (63). The primers and probes are described in Table S1 in the supplemental material. The thermal cycling conditions were initial denaturation at 95°C for 2 min followed by 40 PCR cycles with denaturation at 95°C for 15 s, annealing at 54°C for 1 min, and extension at 72°C for 30 s. Fluorescence measurements were recorded at each annealing step.

10.1128/mBio.01665-21.6 TABLE S1 Strains, plasmids, and PCR primers used in the study. Download Table S1, DOCX file, 0.03 MB. Copyright © 2021 Krishnamoorthy et al. 2021 Krishnamoorthy et al. https://creativecommons.org/licenses/by/4.0/ This content is distributed under the terms of the Creative Commons Attribution 4.0 International license.

### NAD(H), NADP(H), ATP, and glucose-6-phosphate dehydrogenase activity measurements.

One-milliliter samples from aerobic (day 0) and hypoxia-adapted (day 3) culture (from *in vitro* hypoxia experiments described in the legend to Fig. 1B and C) were rapidly harvested and immediately frozen. Cofactor levels in lysed cells were determined using NAD/NADP-Glo and NADP/NADPH-Glo bioluminescent assays (Promega). ATP levels were determined by the BacTiter-Glo microbial cell viability assay (Promega). Additionally, coincubated culture samples (25 ml) were collected and centrifuged. The obtained cell pellets were resuspended in water prior to transfer into a preweighed tube for drying and subsequent cell weight determination. The whole-cell G6PDH activity assay was performed according to the manufacturer’s recommendation (MAK015; Sigma). Whole-cell lysate was prepared by sonicating 5 ml of log-phase culture. G6PDH activity was normalized to the protein concentration measured by the Bradford method (Pierce Coomassie Plus protein assay; Thermo Fisher Scientific).

### Lipid extraction and analysis.

About 125 ml of M. tuberculosis cultures was grown in a roller flask containing tyloxapol-free m7H9 medium containing 0.2% glucose. Lipids were extracted from whole bacterial cells by incubation in chloroform-methanol (1:2, vol/vol) for 2 days at room temperature and then in chloroform-methanol (2:1, vol/vol). Collected organic phases were pooled, washed twice with water, and dried to get crude lipid extracts. Extracted lipids were suspended in chloroform at a final concentration of 20 mg/ml and analyzed by high-performance thin-layer chromatography (HPTLC; Camag). Equal amounts of lipids were spotted on silica gel 60 plates (Merck) with a Camag ATS4 apparatus. The plates were developed using a Camag ADC2 device in various solvent systems (petroleum ether-diethyl ether, 9:1 [vol/vol], for PDIM; chloroform-methanol-water, 65:25:4, for glycolipids). PDIMs were visualized by spraying the plates with 10% phosphomolybdic acid in ethanol, followed by heating, and glycolipids were visualized by spraying the plates with a 0.2% anthrone solution in concentrated sulfuric acid, followed by heating. DIM A and B in enriched fractions were identified by nuclear magnetic resonance spectroscopy.

### Scanning electron microscopy.

Inoculum was prepared with the single colony, and bacterial cells were grown in 10 ml m7H9 medium (supplemented with 0.2% glucose, 0.085% sodium chloride, 0.05% tyloxapol, and 0.5% BSA). Five hundred microliters of log-phase culture (OD_600_, 1.0 to 1.4) was mixed with an equal amount of 4% paraformaldehyde (Electron Microscopy Sciences) and left overnight at 4°C. Subsequently, samples were postfixed with 2.5% glutaraldehyde, contrasted using repeated incubations with 0.5% osmium tetroxide-1% tannic acid, dehydrated with a graded ethanol series, critical point dried, and coated with 3-nm platinum-carbon. Specimens were analyzed in a Leo 1550 field emission scanning electron microscope using the in-lens detector at 20 kV.

### Trypsin digestion and extraction of cell surface-associated proteins.

Trypsin shaving and preparation of cell surface-exposed peptides were performed as described earlier (64). In brief, M. smegmatis mc^2^155 and Δ*mftC* and Δ*mftD* mutant cells were grown (starting OD_600_ of 0.001) in 10 ml m7H9 medium containing 0.2% glucose, 0.5% BSA, and 0.085% sodium chloride with orbital shaking (100 rpm) at 37°C. A 1.5-ml amount of log-phase culture (OD_600_ of 0.8) was harvested and centrifuged at 4,000 × *g* for 5 min at 4°C. PBS-washed (thrice) cells were resuspended in 50 μl of PBS. Forty-five microliters of cell suspension was incubated with 5 μl of trypsin (20 ng/μl; Promega) on a 0.22-μm cellulose acetate centrifuge tube filter by gentle shaking for 15 min at 37°C. To determine possible peptide contaminations from cell lysis, a parallel undigested control was included. Subsequently, the filter tube was centrifuged at 4,000 × *g* for 10 min at 4°C. Filtrate containing peptides (from both undigested and trypsin-digested samples) was again trypsin digested (20 ng/μl) overnight by gentle shaking at 37°C. Samples were acidified with trifluoroacetic acid, and peptides were purified using C_18_ Stage Tips and stored at −20°C until LC-MS analysis.

### Liquid chromatography-mass spectrometry.

All samples were analyzed by LC-MS on a Q-Exactive HF mass spectrometer (Thermo Fisher Scientific) using a 60-min linear gradient delivered by an RSLC Nano 3000 HPLC. Peptides were isolated within a 1.2 *m/z* window and sequenced using a data-dependent acquisition method. MS1 resolution was set to 60,000, and the MS2 resolution was set to 30,000. The MS2 maximum injection time was set to 45 ms, and the automatic gain control (AGC) target was set to 20,000. High-energy collisional dissociation (HCD) normalized collision energy was set to 28. Raw data were processed with MaxQuant (v.1.6.0.1) using default settings. MS/MS spectra were searched against a UniProt M. smegmatis mc^2^155 database. Trypsin/P was set as cleavage specificity. Oxidation (M), protein N-terminal acetylation, and N-terminal glutamate-to-pyroglutamate conversion were set as variable modifications. The minimum peptide length was set to 7 amino acids. Match between runs was enabled. The iBAQ option was enabled to calculate estimates for protein abundance. Downstream data analysis was carried out in Perseus (v.1.6.2.3). Proteins flagged as “potential contaminant,” “reverse,” and “only identified by site” were removed from the data set. iBAQ values were log_2_ transformed, and data were normalized in order to center the median of each sample to the same value. A Student *t* test was applied to determine statistical significance of protein abundance differences between sample groups (fudge factor s0 = 0.1). *P* values were corrected for multiple testing using a permutation-based FDR approach. Protein sequence homology was analyzed using Clustal Omega (65).

### Animal experiments.

All animal studies have been ethically reviewed and approved by the State Office for Health and Social Services, Berlin, Germany. Experimental procedures were carried out in accordance with the European directive 2010/63/EU on Care, Welfare and Treatment of Animals. C57BL/6J, C57BL/6J Ifngr1^−/−^, and C57BL/6J Nos2^−/−^ mice were bred in-house and maintained under specific-pathogen-free conditions. C57BL/6J Ifngr1^−/−^ mice (aged 6 weeks, both sexes) were aerosol infected with approximately 700 CFU of M. tuberculosis strains. C57BL/6J mice (aged 6 to 8 weeks, both sexes) were aerosol infected with approximately 100 CFU of M. tuberculosis H37Rv and mutant derivatives; C57BL/6J Nos2^−/−^ mice were infected with H37Rv and Δ*mftD* strains as previously reported (39). In brief, 6-week-old C57BL/6J Nos2^−/−^ mice (both sexes) were anesthetized (ketamine at 65 mg/kg of body weight, acepromazine at 2 mg/kg, xylazine at 11 mg/kg) and infected with 1,000 CFU of the H37Rv or Δ*mftD* strain in 20 μl PBS administered to the ear dermis. At days 14 and 21 postinfection, each mouse received 0.5 mg of monoclonal anti-tumor necrosis factor alpha antibody (purified from MP6-XT22 cultures) by intraperitoneal injection. Two hours before euthanasia, animals received 60 mg/kg pimonidazole hydrochloride (PIMO) (Hypoxyprobe-1) intraperitoneally to allow for detection of hypoxic regions in organ sections. At dedicated time points, superior, middle inferior, and postcaval lobes from euthanized mice were removed and homogenized in 1 ml PBS-0.05% Tween 80. Serial dilutions of organ homogenates were plated onto m7H11 agar and incubated for 5 weeks at 37°C.

### Staining procedures and histopathology.

Aseptically removed left lung lobe of mice was postfixed in 4% paraformaldehyde for 20 h at room temperature. The tissue was then dehydrated and paraffin embedded (60°C) using a Leica TP 1020 tissue processor. Paraffin blocks were cut at 2 to 3 μm, and sections were mounted and dried on SuperFrost Plus slides (Thermo Fisher Scientific) at 35°C. After dewaxing and rehydration, sections were subjected to hematoxylin and eosin (H&E) staining, or fluorescence staining, to detect PIMO and M. tuberculosis in tissues. Sections were stained with H&E using standard protocols. Central necrosis of lesions was defined as a lighter pink region, indicating tissue consolidation, surrounded by granulomatous inflammatory infiltrates. A researcher blind to the study groups scored at least four individual stained sections of each organ in study groups of five mice per time point.

Immunofluorescence microscopy on mouse lung and image acquisition and processing were performed as previously reported (60). Slides were incubated with 10 mg/ml DNA stain Hoechst 33342 (1:5,000) for 5 min and rinsed with water before mounting with Mowiol. The procedure used rabbit anti-M. tuberculosis antibody (Abcam; ab905; 1:1,000) with secondary detection with donkey anti-rabbit immunoglobulin G heavy and light chain Cy3 (Jackson Immunoresearch; 711-166-152; 1:200) and fluorescein isothiocyanate (FITC)-conjugated mouse anti-PIMO as primary antibody (included in Hypoxyprobe-1 kit; 1:50), with secondary detection of PIMO with goat anti-FITC (Abcam; ab19224; lot no. GR175456-35; 1:100, incubated for 2 h/room temperature) followed by donkey anti-goat Alexa Fluor 488 (Jackson Immunoresearch; 705-546-147; 1:200).

### Statistical analysis.

GraphPad Prism 7.03 software was used for statistical analyses. *P* values of less than 0.05 were considered statistically significant. Bacterial growth rate during the exponential growth phase in liquid culture experiments was estimated according to the Malthusian growth model (66). Further details are shown in figure legends.

### Data availability.

The mass spectrometry proteomics data have been deposited to the ProteomeXchange Consortium via the PRIDE partner repository (67) with the data set identifier PXD023045.
