# Supplementary figures and images for: Role of Premycofactocin Synthase in Growth, Microaerophilic Adaptation, and Metabolism of Mycobacterium tuberculosis
Source: mBio. 2021 Jul 27;12(4):e01665-21. doi: 10.1128/mBio.01665-21 (PMC8406134; doi:10.1128/mBio.01665-21)

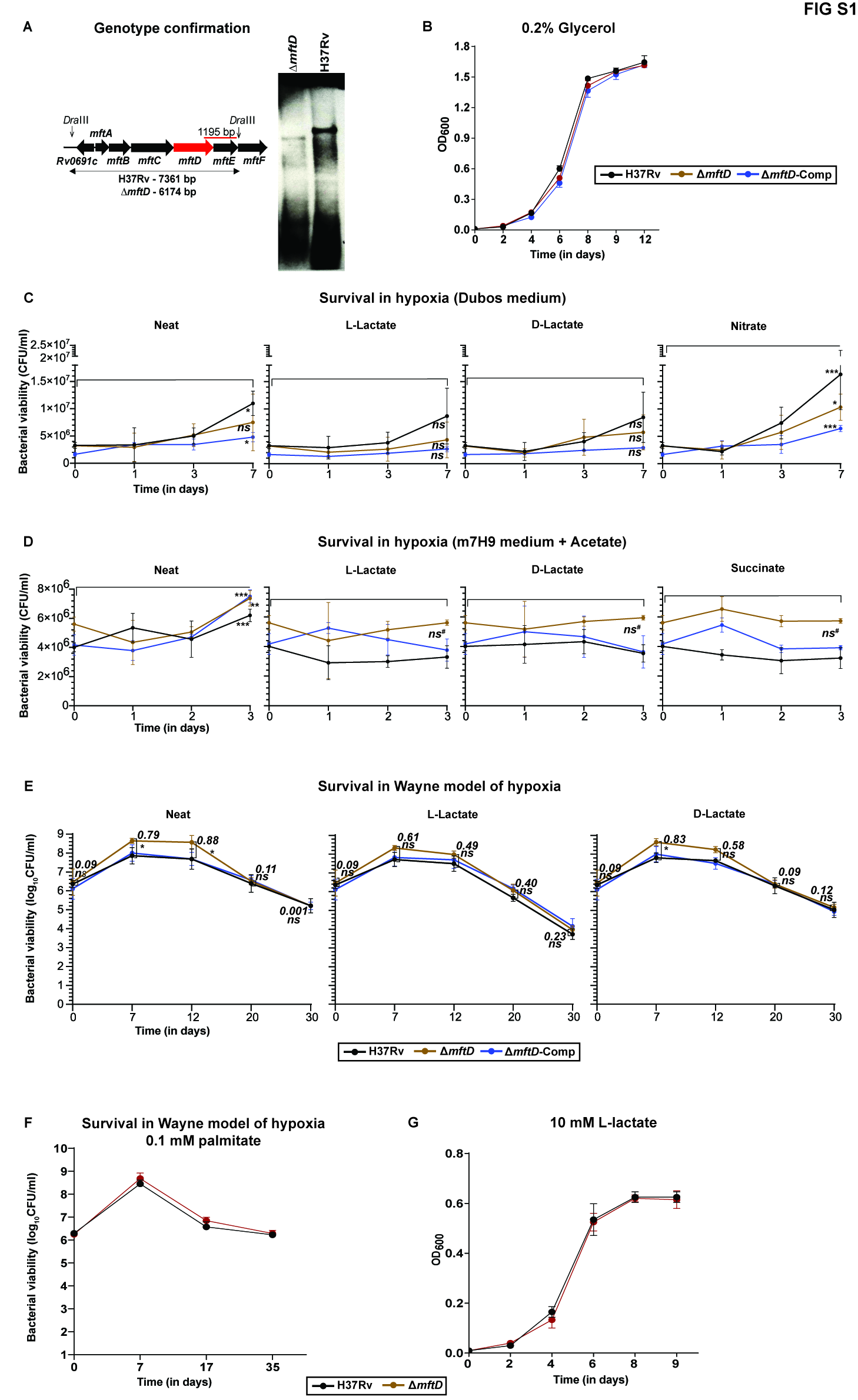

Supplement: FIG S1 [file mbio.01665-21-sf001.tif]

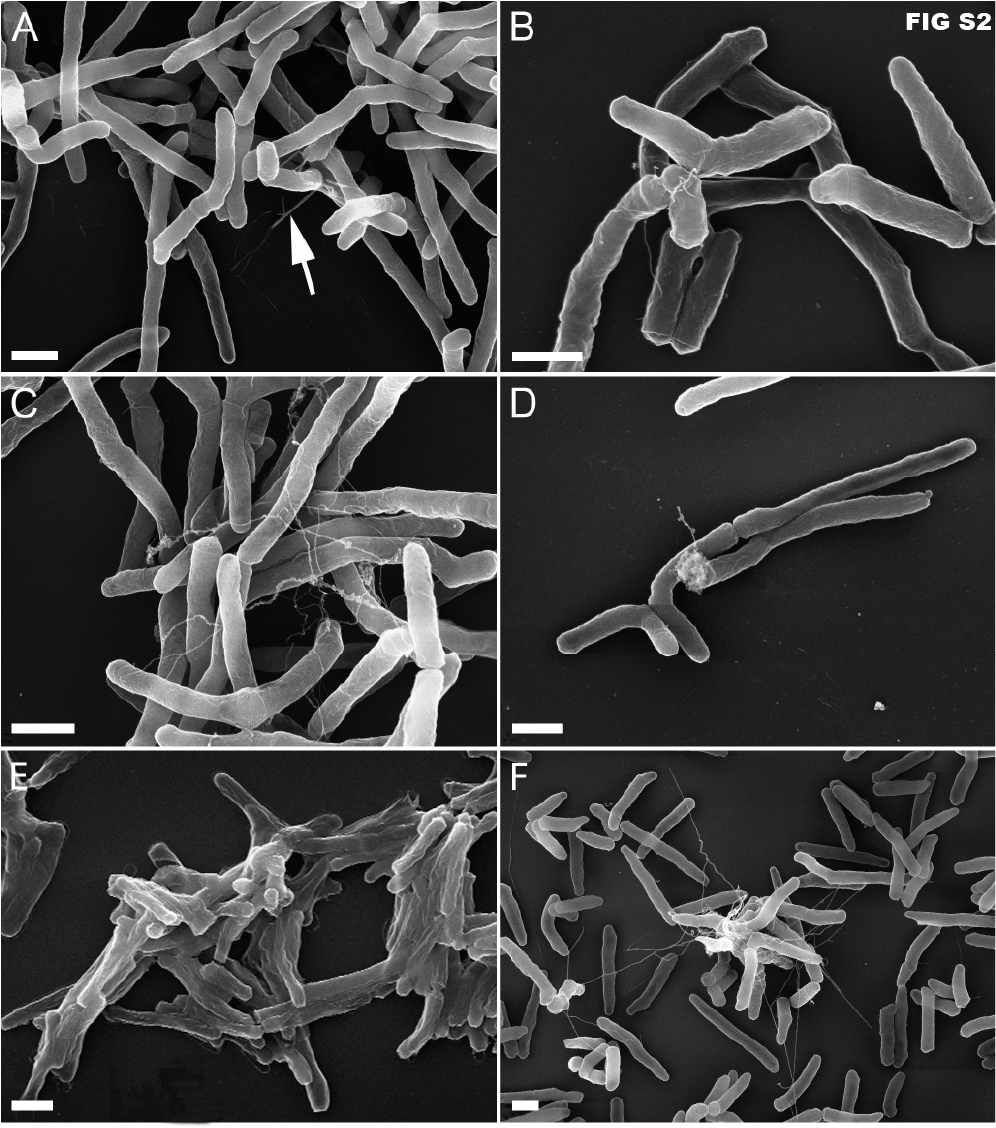

Supplement: FIG S2 [file mbio.01665-21-sf002.tif]

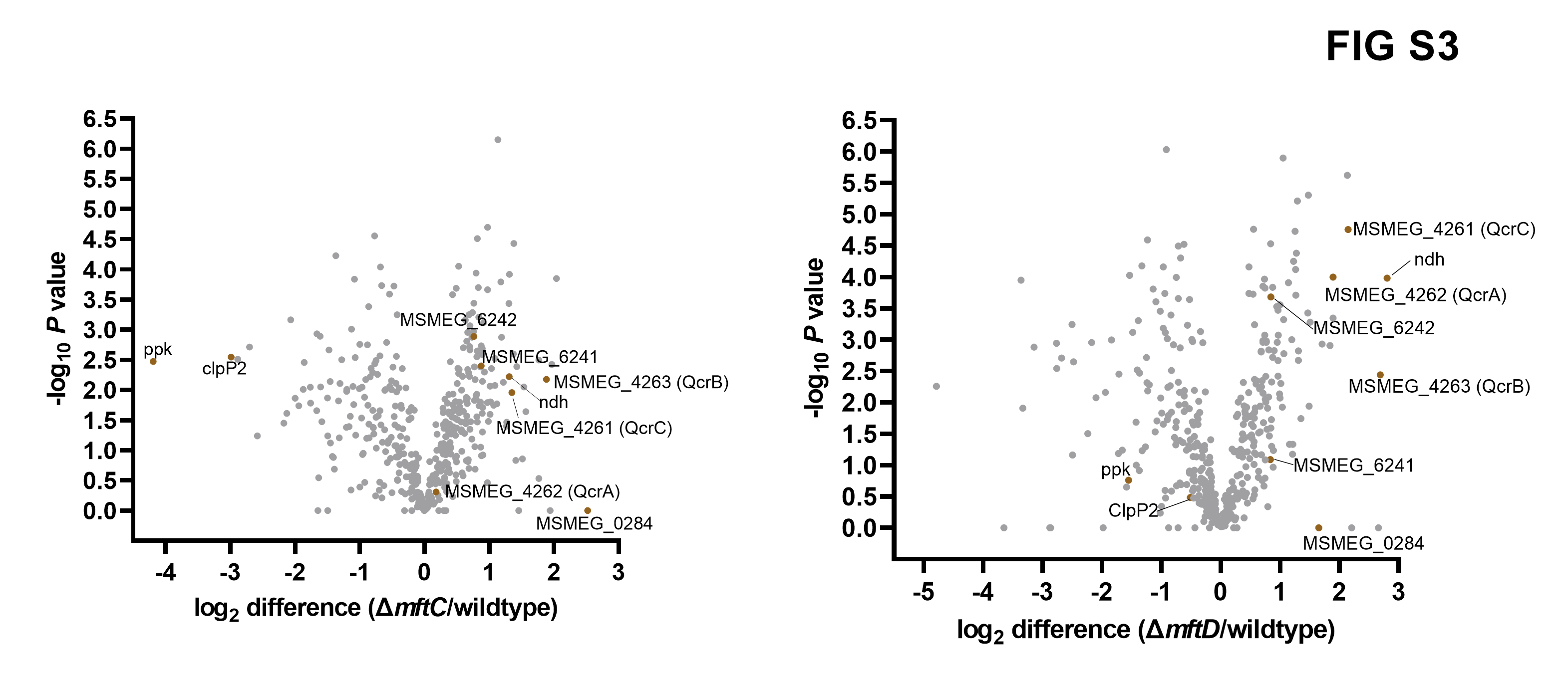

Supplement: FIG S3 [file mbio.01665-21-sf003.tif]

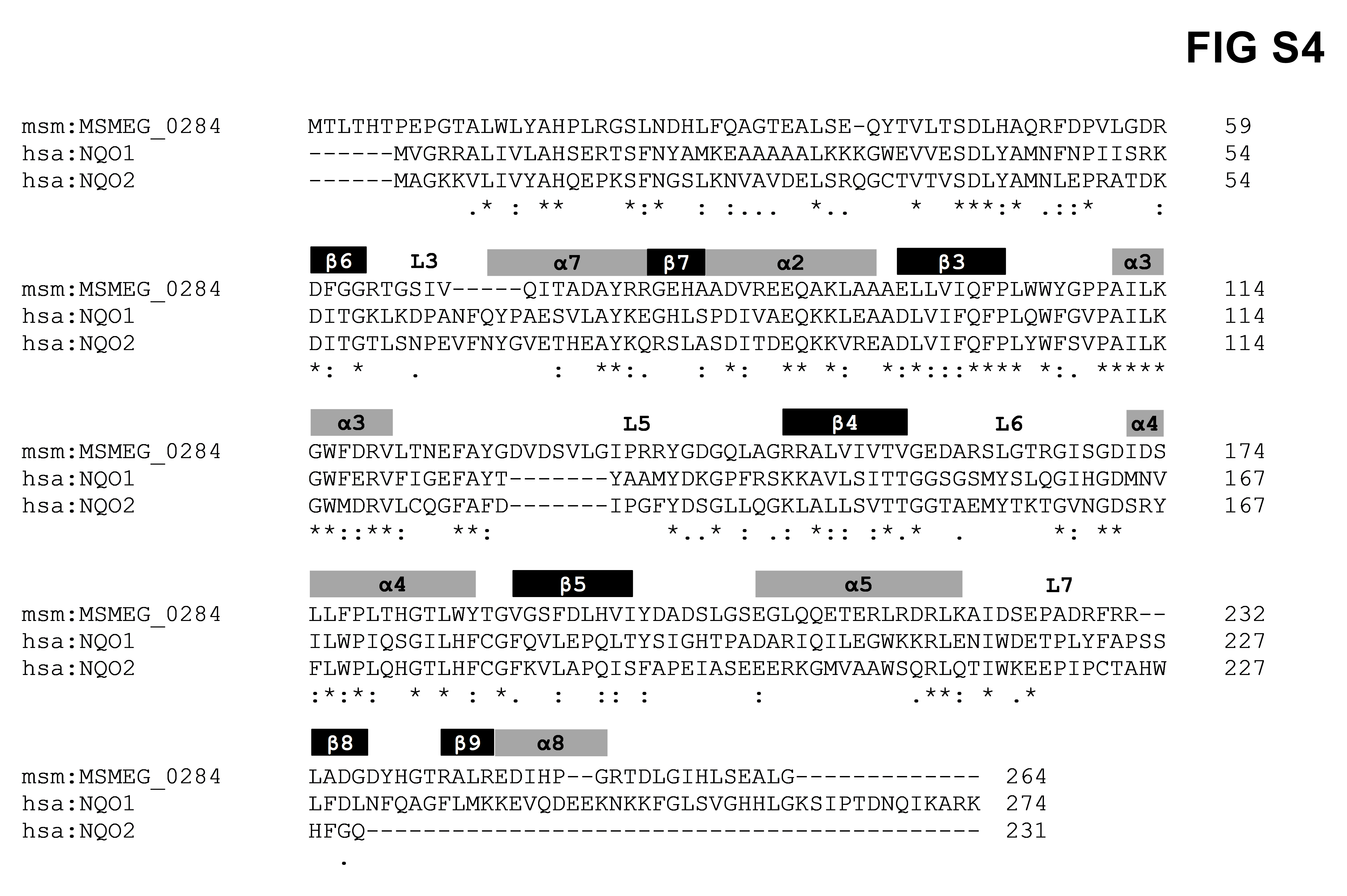

Supplement: FIG S4 [file mbio.01665-21-sf004.tif]

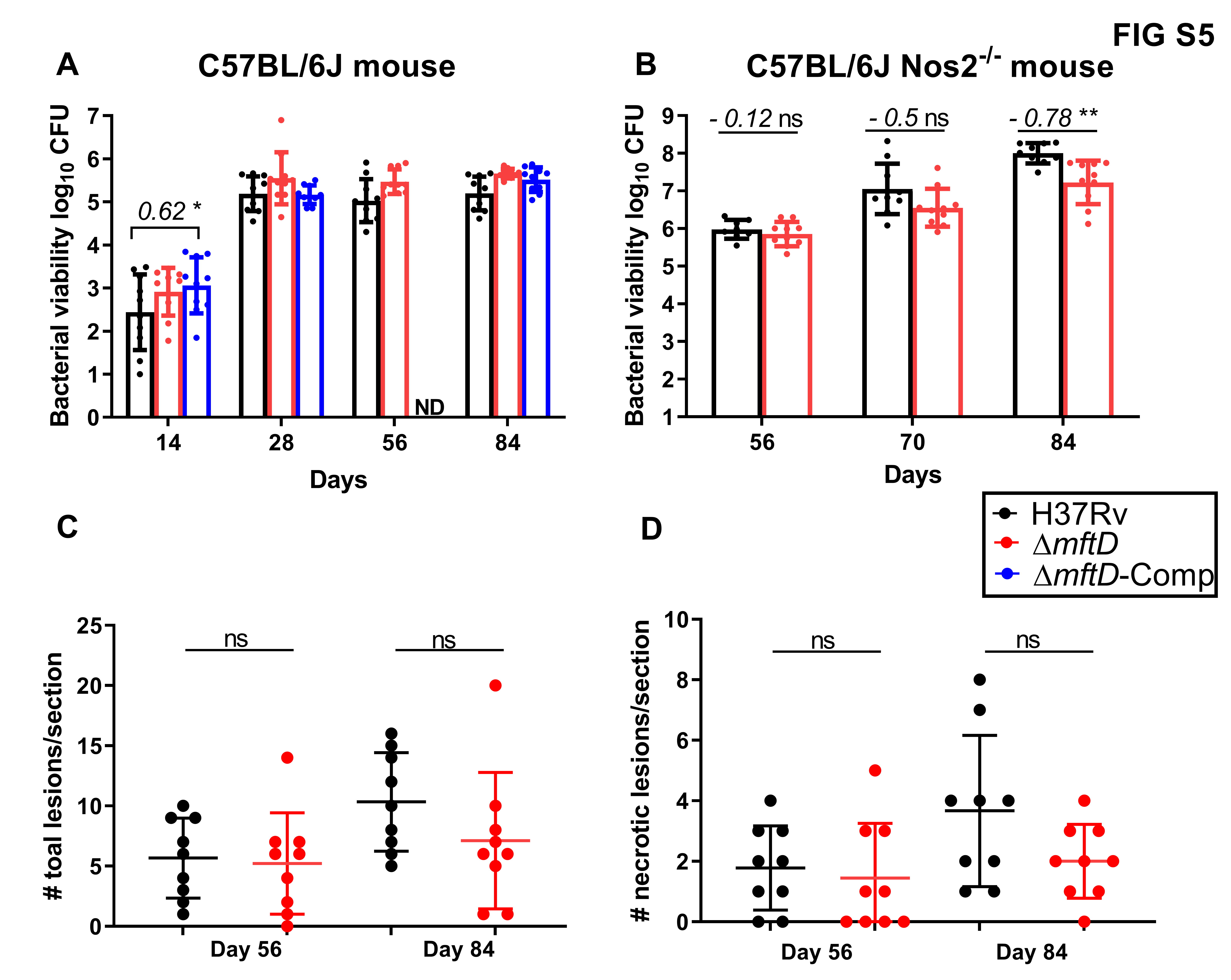

Supplement: FIG S5 [file mbio.01665-21-sf005.tif]
