# Supplementary material for: Role of Premycofactocin Synthase in Growth, Microaerophilic Adaptation, and Metabolism of Mycobacterium tuberculosis
Source: mBio. 2021 Jul 27;12(4):e01665-21. doi: 10.1128/mBio.01665-21 (PMC8406134; doi:10.1128/mBio.01665-21)
Supplement: TABLE S1 [file mbio.01665-21-st001.docx]

| Gopinath Krishnamoorthy, Peggy Kaiser, Patricia Constant, Ulrike Abu Abed, Monika Schmid, Christian K. Frese, Volker Brinkmann, Mamadou Daffe, Stefan H. E. Kaufmann. Role of Pre-mycofactocin synthase in growth, microaerophilic adaptation, and metabolism of *Mycobacterium tuberculosis*.  TABLE S1. Strains, plasmids, PCR primers used in the study | | |
| --- | --- | --- |
| Strain | **Description** | **Source** |
| *Escherichia coli* |  |  |
| DH5α | F- *φ80lacZΔM15* Δ(*lacZYA*-*argF*) U169 *recA1* *endA1* *hsdR17*(rk^-^, mk^+^) *phoAsupE44* *thi*-1 *gyrA96* *relA1* λ- | Invitrogen |
|  |  |  |
| *M. smegmatis* |  |  |
| mc^2^155 | High-frequency transformation mutant of *M. smegmatis* mc^2^6; ATCC^®^ 700084^™^ | (1) |
| ∆*mftA* | in-frame deletion mutant lacking 153 bp region of *mftA* (*MSMEG_1421*) | (2) |
| ∆*mftB* | in-frame deletion mutant lacking 300 bp region of *mftB* (*MSMEG_1422*) | (2) |
| ∆*mftC* | in-frame deletion mutant lacking 1169 bp region of *mftC* (*MSMEG_1423*) | (2) |
| ∆*mftD* | in-frame deletion mutant lacking 1196 bp region of *mftD* (*MSMEG_1424*) | (2) |
| ∆*mftE* | in-frame deletion mutant lacking 649 bp region of *mftE* (*MSMEG_1425*) | (2) |
| ∆*mftF* | in-frame deletion mutant lacking 1403 bp region of *mftF* (*MSMEG_1426*) | (2) |
| ∆*mftC*-Comp | Complemented *mftC* mutant carrying pMCpAINT::*mftC* at the *attB* site; Kan^R^ | (2) |
|  |  |  |
| *M. marinum* |  |  |
| *M. marinum* | Laboratory stock of *M. marinum* M strain |  |
| ∆*mftD* | in-frame deletion mutant lacking 1167 bp region of *mftD* (*MMAR_1022*) | (2) |
|  |  |  |
| *M. tuberculosis* |  |  |
| H37Rv | Laboratory stock of *M. tuberculosis* strain ATCC^®^ 27294^™^ |  |
| ∆*mftD* | in-frame deletion mutant lacking 1185 bp region of *mftD* | This study |
| ∆*mftD*-Comp | Complemented *mftC* mutant carrying 3351 bp region of the *M. tuberculosis H37Rv* chromosome containing sequences of *Rv0691c* to *Rv0694* in the integrative vector pMCpAINT; Km^R^ | This study |
|  |  |  |
| Plasmids | **Description** | **Source** |
| p2NIL | *E. coli* cloning vector; (Kanamycin resistant - Km^R^) | (3) |
| pGOAL19 | Plasmid carrying *hyg, lacZ* and *sacB* genes as a *Pac*I cassette; (Hygromycin resistant - Hyg^R^; Ampicillin resistant - Ap^R^) | (4) |
| pMCpAINT | *E. coli*–Mycobacterium integrating shuttle vector; Km^R^ | (5) |
| p19∆*mftD* | *mftD* knockout vector. p2NIL sub clone carrying ∆*Rv0694* gene – fusion of PCR products upstream (1194 bp) and downstream (960 bp) of *Rv0694* and *hyg-lacZ-sacB* cassette from pGOAL19; Km^R^ Hyg^R^ | This study |
| pMCpAINT::*mftD* | *mftD* complementation vector. pMCpAINT harbouring PCR product of 3351 bp region of the *M. tuberculosis* H37Rv chromosome containing the *Rv0691c* to *Rv0694* nucleotide sequences | This study |
| p2NIL (Addgene plasmid # 20188) and pGOAL19 (Addgene plasmid # 20190) was a gift from Tanya Parish. pMCpAINT was a gift from Digby Warner. | | |

| **Oligonucleotides** | **Sequence (5´-3´)** | **Amplicon properties/ region targeted** |
| --- | --- | --- |
| **Oligonucleotides used for knockout vector construction^a^** | | |
| Rv0694 (*mftD*) UP F | *ggcgg***aagctt**atgacgagccccgt | Forward and reverse primers used to PCR amplify 1167 bp + 3 bp from 5′ end of *mftD* (*Rv0694*) |
| Rv0694 (*mftD*) UP R | *ggcgg***ctcgag**ccacggctacaccggactttc |  |
| Rv0694 (*mftD*) DOWN F | *ggcgg***ctcgag**tagccggatgttgagc | Forward and reverse primers used to PCR amplify 931 bp + 3 bp from 3′ end of *mftD* (*Rv0694*) |
| Rv0694 (*mftD*) DOWN R | *ggcgg***ggtacc**tcatgtcaacatcccgtcgggc |  |
| **Oligonucleotides used for genetic complementation vector construction^a^** | | |
| Rv0694_Compl. New F | *ggcgg***aagctt**cttgtagctgtgcaaggtgggtgc | Forward and reverse primers used to PCR amplify 3351 bp region of the *M. tuberculosis* H37Rv chromosome containing sequences of *Rv0691c* to *Rv0694*. |
| Rv0694_Compl. New R | *ggcgg***aagctt**tcaacatccggctaaacgtcccgtcggg |  |
| **^a^GC-clamp sequences (non-H37Rv) are italicized; Restriction sites are shown in bold.** | | |
| **Oligonucleotides used for PCR-based genotyping** | | |
| Rv0694 (*mftD*) Screen F: | ctcgtcgctgtttcgcgagctccgggagc | Forward and reverse primers used for PCR-based  genotyping of the *mftD*. ∆*mftD*: 612 bp; WT: 1797 bp. |
| Rv0694 (*mftD*) Screen R: | cggtggaccccagcgggatcactatcgacg |  |
| **Oligonucleotides used for probe generation for southern blot analysis** | | |
| *Rv0694* (*mftD*) Southern F | ccggtgcaatccgccgagcagccgaagcggcacc | 770 bp amplicon used as a probe for genotyping ∆*mftD* |
| *Rv0694* (*mftD*) Southern R | gggccgacggcgctcaaccacaccgcaccac |  |
| **Oligonucleotides used for qRT-PCR** | | |
|  | Forward and reverse | Probe |
| ***Rv0693*** (*mftC*) | cccattcgccattcatga  cgagttcttccagacattttg | 6-FAM-atccgccgtcggacaacac-MGB-Q500 |
| ***Rv0694*** (*mftD*) | gatccccgaagagatgaac  cttggcgaacttccacaa | 6-FAM-aatccgccgtcggacaacac-MGB-Q500 |
| ***hspX*** | ccgagcgcaccgagcagaag ggtggccttaatgtcgtcctcgtc | 6-FAM-gctccccttcgttcgcacggtgtcggagcc-MGB-Q500 |
| ***sigA*** | atgacgacgaggagatcgctga  tccttgcgtgcttgacgcag | 6-FAM-cgcgatccggtgatttcgtctgggatggatcgcg-MB-Q500 |

1. Snapper, S. B., R. E. Melton, S. Mustafa, T. Kieser, and W. R. Jacobs, Jr**.** 1990. Isolation and characterization of efficient plasmid transformation mutants of *Mycobacterium smegmatis*. Mol Microbiol **4:**1911-9.
2. Gopinath Krishnamoorthy, Peggy Kaiser, Laura Lozza, Karin Hahnke, Hans-Joachim Mollenkopf, Stefan H. E. Kaufmann. 2019. Mycofactocin Is Associated with Ethanol Metabolism in Mycobacteria. mBio. 10(3): e00190-19.
3. Parish T, Stoker NG. 2000. Use of a flexible cassette method to generate a double unmarked *Mycobacterium tuberculosis tlyA* *plcABC* mutant by gene replacement. Microbiology **146**:1969-75.
4. Parish T, Stoker NG. 2000. Use of a flexible cassette method to generate a double unmarked *Mycobacterium tuberculosis tlyA* *plcABC* mutant by gene replacement. Microbiology **146**:1969-75.
5. Warner DF, Ndwandwe DE, Abrahams GL, Kana BD, Machowski EE, Venclovas Č, Mizrahi V. 2010. Essential roles for *imuA*′-and *imuB*-encoded accessory factors in DnaE2-dependent mutagenesis in *Mycobacterium tuberculosis*. Proceedings of the National Academy of Sciences **107**:13093-13098.
